# Arterial stiffness is an independent predictor for risk of mortality in patients with type 2 diabetes mellitus: the REBOUND study

**DOI:** 10.1186/s12933-020-01120-6

**Published:** 2020-09-22

**Authors:** Jeong Mi Kim, Sang Soo Kim, In Joo Kim, Jong Ho Kim, Bo Hyun Kim, Mi Kyung Kim, Soon Hee Lee, Chang Won Lee, Min Chul Kim, Jun Hyeob Ahn, Jinmi Kim, for the Relationship between Cardiovascular disease and Brachial-ankle Pulse Wave Velocity (baPWV) in Patients with Type 2 Diabetes (REBOUND) Study Group

**Affiliations:** 1 Division of Endocrinology and Metabolism, Department of Internal Medicine, Biomedical Research Institute, Pusan National University Hospital, 179, Gudeok-ro, Seo-gu, Busan, 49241 South Korea; 2 Department of Internal Medicine, Inje University Haeundae Paik Hospital, Inje University College of Medicine, Busan, South Korea; 3 Department of Internal Medicine, Inje University Busan Paik Hospital, Inje University College of Medicine, Busan, South Korea; 4 Department of Internal Medicine, Busan St. Mary’s Hospital, Catholic University of Pusan, Busan, South Korea; 5 Department of Internal Medicine, Ilsin Christian Hospital, Busan, South Korea; 6 Department of Internal Medicine, Good Moonhwa Hospital, Busan, South Korea; 7 Department of Biostatistics, Clinical Trial Center, Biomedical Research Institute, Pusan National University Hospital, Busan, South Korea; 8 Present Address: Department of Internal Medicine, Isam Hospital, Busan, South Korea

**Keywords:** Vascular stiffness, Brachial-ankle pulse wave velocity, Type 2 diabetes mellitus, Mortality

## Abstract

**Background:**

This study aimed to evaluate the benefit of brachial-ankle pulse wave velocity (baPWV) as a noninvasive marker of arterial stiffness for the prediction of all-cause and cause-specific mortality in patients with type 2 diabetes.

**Methods:**

This multicenter prospective observational study analyzed 2308 patients with type 2 diabetes between 2008 and 2018. The patients were categorized according to the quartiles of baPWV. Cause of mortality was determined using death certificates and patient clinical records. We estimated proportional mortality rates from all causes, cardiovascular, cancer, and other causes among adults with diabetic status according to their baPWV. Cox regression models were used to estimate hazard ratios (HRs).

**Results:**

There were 199 deaths (8.6%) in the study population during a median follow-up duration of 8.6 years. When baPWV was assessed as quartiles, a significantly higher risk of all-cause mortality (HR = 5.39, *P* < 0.001), cardiovascular-mortality (HR = 14.89, *P* < 0.001), cancer-mortality (HR = 5.42, *P* < 0.001), and other-cause mortality (HR = 4.12, *P* < 0.001) was found in quartile 4 (Q4, ≥ 1830 cm/s) than in quartiles 1–3 (Q1–3). Adding baPWV to baseline model containing conventional risk factors such as age, sex, diabetes duration, body mass index, glycated hemoglobin, systolic blood pressure, glomerular filtration rate, smoking, and insulin improved the risk prediction for all-cause (net reclassification index (NRI) = 49%, *P* < 0.001) and cause-specific (cardiovascular NRI = 28%, *P *= 0.030; cancer NRI = 55%, *P* < 0.001; other-cause NRI 51%, *P* < 0.001) mortality.

**Conclusion:**

This long-term, large-scale, multicenter prospective observational cohort study provide evidence that increased arterial stiffness, as measured by baPWV, predicts the risk of all-cause and cause-specific mortality in type 2 diabetes, supporting the prognostic utility of baPWV.

*Trial registration* Clinical Research Information Service (CRIS), KCT 0005010. Retrospectively Registered May 12, 2020. https://cris.nih.go.kr/cris/search/search_result_st01.jsp?seq=16677

## Background

Diabetes has reached epidemic proportions globally. Current estimates by the International Diabetes Federation suggest that 451 million people had diabetes in 2017 and will reach 693 million by 2045 [1]. Given the substantial rise in the prevalence of diabetes, its related morbidity and mortality contribute to a catastrophic socioeconomic burden [2]. Thus, it is imperative to have comprehensive estimates on the causes of death from diabetes to enable planning for the allocation of apposite health resources to combat this tragedy.

The presence of diabetes doubles or quadruples the risk of a diverse range of cardiovascular (CV) diseases, and the life expectancy of these patients becomes shorter than that of individuals without diabetes [3–5]. Reduced life expectancy for individuals with diabetes is strongly associated with CV death, but cancer and other pathologies are also highlighted as the main risk factors leading to death in diabetes [6, 7]. Emerging evidence demonstrating a marked decrease in CV death turns the spotlight onto other causes of death in individuals with diabetes [8, 9]. In particular, the excess death rate from various types of cancer in individuals with diabetes exposes the vulnerability of patients with diabetes [10]. Indeed, diabetes and cancer share robust common risk factors that contribute to death [11].

Arterial stiffness is closely associated with atherosclerotic risk factors and may predict the short- and long-term prognosis for CV events, especially in individuals with diabetes [12, 13]. It can be assessed by pulse wave velocity (PWV), a simple, noninvasive, and widely used tool in clinical practice. Brachial-ankle pulse wave velocity (baPWV), calculated as the distance between the brachial and the tibial artery divided by the pulse wave transit time between these two arteries, has been proposed as a surrogate end point for CV disease (CVD) [14]. Many studies suggest that an abnormal baPWV is an indicator of the degree of arteriosclerosis and is associated with adverse CV outcomes in subjects at high risk of CV events, including individuals with diabetes [15–17]. However, knowledge of the prognostic impact of arterial stiffness for cause-specific mortality is still limited.

In this study, we aimed to investigate the predictive ability of arterial stiffness for all-cause and cause-specific mortality in a large prospective cohort with type 2 diabetes.

## Methods

### Study design and population

This study assessed subjects enrolled in the Relationship between Cardiovascular Disease and Brachial-ankle Pulse Wave Velocity (baPWV) in Patients with Type 2 Diabetes (REBOUND) Study. The REBOUND study is a multicenter prospective observational study to assess the association between baPWV and CVD in patients with type 2 diabetes. A detailed description of the design has been published previously [18, 19]. Briefly, the REBOUND study was conducted at eight general and teaching hospitals in Busan, Korea. A total of 3058 Korean patients with type 2 diabetes were enrolled consecutively at outpatient clinics between June 2008 and December 2010. The inclusion criteria for patients were (i) age > 30 years and (ii) measurement of baseline baPWV. The exclusion criteria were (i) type 1 diabetes mellitus, (ii) an ankle brachial index (ABI) of < 0.9, (iii) severe symptoms and/or signs of CVD (i.e., shortness of breath, constant dizziness, and chest pain), and (iv) hospitalization within the previous 3 months due to acute myocardial infarction, stroke, or heart failure.

**Fig. 1:**
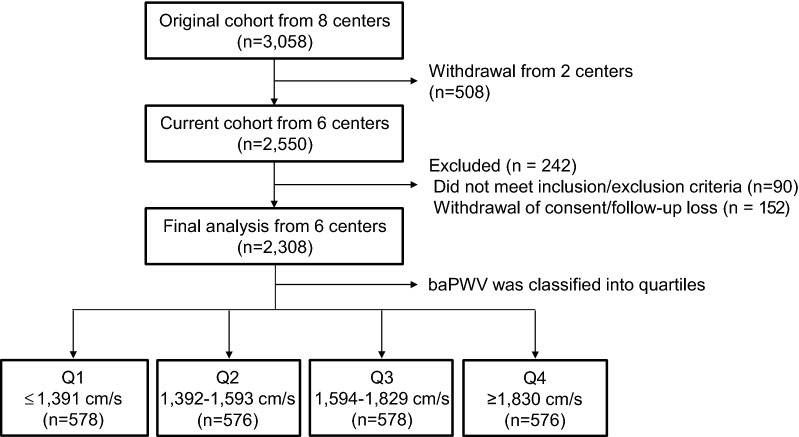
Flow chart of the current cohort. *baPWV* brachial-ankle pulse wave velocity

For the analysis presented here, two hospitals were excluded from the original eight hospitals due to the principal investigator’s relocation. A total of 2550 subjects from six hospitals (Busan St. Mary’s Hospital, Ilsin Christian Hospital, Inje University Busan Paik Hospital, Inje University Haeundae Paik Hospital, Good Moonhwa Hospital, and Pusan National University Hospital) were followed up at each clinic until the date of death or February 2018, whichever occurred first. The best treatment according to standard guidelines at each outpatient clinic was followed. Furthermore, 2550 subjects from the six hospitals had measurement of their baseline baPWV, as a noninvasive marker of arterial stiffness, based on the procedures of the inpatient or outpatient endocrinology departments of each hospital. Of these 2550 patients, 90 who did not meet the inclusion criteria or meet any of the exclusion criteria were excluded. Moreover, 152 patients were excluded from the analysis due to withdrawal of consent or lost to follow-up. Thus, a total of 2308 patients were included in the analysis (Fig. 1).

### Measurement of baPWV

All study patients continued taking their regular medication during the study. Measurement of baPWV took place with the patient in supine position after at least 5-min rest. Left and right baPWVs were simultaneously measured using an automatic waveform analyzer (from ABI/PWV, VP-2000, Colin CO. Ltd, Komaki, Japan) according to the manufacturer’s recommendations and a previous validated method [20]. The higher value was defined as the maximum baPWV, and this value was used for each individual for all analyses in this study.

### Other variables

The subjects fasted for at least 8 h before investigators measured their fasting glucose level, glycated hemoglobin, serum lipid, serum insulin, C-peptide, high sensitivity C-reactive protein (hsCRP), aspartate aminotransferase, alanine aminotransferase, γ-glutamyl transferase, serum and urine creatinine, and urine microalbumin. The estimated glomerular filtration rate (eGFR) levels were calculated from the serum creatinine levels using the Chronic Kidney Disease Epidemiology Collaboration equation [21].

Baseline and follow-up examinations included a physical examination, laboratory testing, medical history, and an in-person interview to collect information regarding medical conditions. Diabetic retinopathy was detected during an eye examination that included fundus photography (also known as ophthalmoscopy). Diabetic neuropathy was diagnosed based on symptoms, medical history, and physical examination. Nephropathy was defined as an eGFR < 60 mL/min/1.73 m^2^ or high levels of albumin in the urine (≥ 30 mg/g). Participants were categorized as current and former or never smokers according to their smoking history. CVD includes coronary heart disease, cerebrovascular disease, peripheral arterial disease, rheumatic heart disease, congenital heart disease, deep vein thrombosis, and pulmonary embolism. Treatment modalities of study population were divided into antihyperglycemic, antihypertensive, antidyslipidemic, and antiplatelet agents.

### Clinical end points

Participants with no medical record of death were deemed as alive and were censored at the end of follow-up (February 2018). For the analysis of specific cause-related mortality, study populations with other leading causes of death were censored at the date of death. This was confirmed by a death certificate. The cause of mortality was classified according to the International Classification of Disease (ICD)-10 codes: CV (I00-99), cancer (C00-97), and other causes (codes other than those mentioned above). Other causes of mortality were infection, respiratory disease, kidney disease, and trauma.

### Statistical analyses

To analyze the association of baPWV with mortality, the study populations were divided into four groups according to the quartiles of baseline baPWV. Continuous variables are presented as mean ± standard deviation (SD) or median values with their interquartile ranges, and categorical variables are expressed as number and percentage. After determining the normality of the data, normally distributed data was compared with one-way ANOVA while non-normally distributed data was compare with Kruskal–Wallis test. Chi square test was used to evaluate the categorical variables. A Cox proportional hazards regression model for all-cause and cause-specific mortality was used to estimate hazard ratios (HRs) and 95% confidence intervals (CIs). In multivariate models, adjustments were performed for age, sex, body mass index (BMI), diabetes duration, glycated hemoglobin, systolic blood pressure (SBP), GFR, smoking, and insulin. We simply treated all competing events as though the individuals were right censored at the time the competing event occurred. Cumulative incidence curves of all-cause and cause-specific mortality are calculated using the Kaplan–Meier approach without accounting for competing risk events. To assess the improvement in risk prediction for all-cause and cause-specific mortality by adding baPWV as risk factor in the baseline model, we calculated area under the receiver-operating characteristic curve (AUC), Net reclassification index (NRI) and integrated discrimination improvement (IDI) by comparing these two models. All statistical analyses were performed using R Statistical package version 4.0.2 (https://www.R-project.org), and for all analyses, a *P* value < 0.05 was considered statistically significant.

## Results

### Clinical characteristics of the patients

A total of 2308 patients with type 2 diabetes were included in the study. Table 1 shows the baseline characteristics of the study population according to the quartiles of baseline baPWV. The mean age of the total study population was 58.5 (± 11.0) years, 44% of the patients were men, and the mean baseline baPWV was 1645 cm/s. Over the quartiles of baPWV, age, disease duration, SBP, and diabetic microvascular complications (diabetic retinopathy and neuropathy) were significantly increased, while glycated hemoglobin, diastolic blood pressure, lipid profile, hsCRP, and serum creatinine remained the same. Antihypertensive drug use increased across the quartiles, and male sex was negatively associated with baPWV.

**Table 1:** Baseline characteristics of study participants, categorized according to the quartiles of baPWV

Characteristics	Total	baPWV quartiles, cm/s				P value
		Q1 (–1391)	Q2 (1392–1593)	Q3 (1594–1829)	Q4 (1830–)	
N	2308	578	576	578	576	
Age (years)	58.5 ± 11.0	52.1 ± 10.6	56.3 ± 9.6	60.1 ± 9.6	65.5 ± 9.7	< 0.001
Sex, male (%)	1005 (44)	281(49)	276(48)	237(41.0)	211(37)	< 0.001
Smoking (%)	523 (23)	147 (25)	148 (26)	124 (22)	104 (18)	< 0.001
Body weight (kg)	64.8 ± 11.0	67.4 ± 12.4	65.9 ± 10.4	64.02 ± 10.3	61.7 ± 10.0	< 0.001
BMI (kg/m^2^)	24.9 ± 3.4	25.2 ± 3.7	25.1 ± 3.2	24.9 ± 3.3	24.6 ± 3.3	0.020
Diabetes duration (years)	8.7 ± 7.1	6.7 ± 5.7	7.9 ± 6.3	9.0 ± 7.1	11.3 ± 8.3	< 0.001
HbA1c (%)	7.2 (6.5–8.5)	7.2(6.4–8.3)	7.1 (6.5–8.3)	7.2 (6.5–8.5)	7.4 (6.7–8.7)	0.004
SBP, mmHg	130 (120–140)	120 (112–129)	128 (120–136)	130 (121–140)	138 (130–151)	< 0.001
DBP, mmHg	80 (72–85)	75 (70–81)	80 (73–85)	80 (73–85)	80 (74–88)	< 0.001
Total cholesterol (mg/dL)	174 (148–202)	174 (148–203)	176 (149–204	173 (148–203)	172 (148–201	0.525
Triglyceride (mg/dL)	124 (89–181)	125 (87–178)	124 (88–191)	125 (89–183)	124 (94–175)	0.902
LDL cholesterol (mg/dL)	93 (74–117)	96 (74–120)	93 (76–115)	90 (71–116)	94 (72–117)	0.239
HDL cholesterol (mg/dL)	46 (39–55)	47 (40–54)	47 (41–56)	46 (39–55)	46 (38–54)	0.024
hsCRP	0.2 (0.1–0.80)	0.2 (0.1–0.7)	0.2 (0.1–0.6)	0.2 (0.1–1.0)	0.2 (0.1–1.2)	0.038
Serum creatinine (mg/dL)	0.9 (0.7–1.1)	0.9 (0.7–1.0)	0.9 (0.7–1.0)	0.9 (0.7–1.0)	0.9 (0.8–1.1)	< 0.001
Maximum baPWV (cm/s)	1645 ± 347	1278 ± 84	1489 ± 57	1701 ± 67	2114 ± 292	< 0.001
Diabetic retinopathy (%)	383 (19)	66 (13)	68 (14)	104 (21)	145 (31)	< 0.001
Diabetic nephropathy (%)	218 (10)	30 (5)	27 (5)	57 (10)	104 (19)	< 0.001
Diabetic neuropathy (%)	959 (43)	197 (35)	211 (38)	249 (45)	302 (55)	< 0.001
Insulin	695 (30)	152 (26)	153 (27)	167 (29)	223 (39)	< 0.001
Antihypertensive drugs	1480 (64)	282 (49)	345 (60)	414 (72)	439 (76)	< 0.001
Lipid-lowering drugs	1462 (63)	344 (60)	360 (63)	387 (67)	371 (64)	0.061
Antiplatelet drugs	1321 (57)	270 (47)	303 (53)	381 (66)	367 (64)	< 0.001

Data are presented as number (percentage), mean ± SD, or median (interquartile range)

*BMI* body mass index, *HbA1c* glycated hemoglobin, *SBP* systolic blood pressure, *DBP* diastolic blood pressure, *LDL* low-density lipoprotein, *HDL* high-density lipoprotein, *hsCRP* high sensitivity C-Reactive Protein, *baPWV* brachial-ankle pulse wave velocity

### Association of baPWV with all-cause and cause-specific mortality

Table 2 presents the results of the univariate and multivariate analyses of all-cause and cause-specific mortality according to the quartiles of baPWV. A total of 199 deaths (9%) occurred during a median follow-up period of 8.6 (8.2–9.0) years. In the univariate analysis, the group with the highest quartile of baPWV (Q4) had a statistically significant higher risk of all-cause mortality (HR 5.39, 95% CI 3.44–8.44, *P* < 0.001), CV-mortality (HR 14.89, 95% CI 3.54–62.61, *P *< 0.001), cancer-mortality (HR 5.42, 95% CI 2.25–13.02, *P* < 0.001), and other-cause mortality (HR 4.12, 95% CI 2.33–7.28, *P* < 0.001) than all other three quartiles of baPWV (Q1–3). In the multivariate analysis, after adjusting for confounding factors such as age, sex, diabetes duration, BMI, glycated hemoglobin, SBP, GFR, smoking, and insulin, the group with highest quartile of baPWV (Q4) still had a significantly higher risk of all-cause mortality (HR 2.55 95% CI 1.49–4.35, *P* = 0.001), CV-mortality (HR 5.57 95% CI 1.19–26.18, *P* = 0.030), and cancer-mortality (HR 4.35, 95% CI 1.57–12.03, *P* = 0.005) with the exception of other-cause mortality (HR 1.59, 95% CI 0.78–3.26, *P* = 0.207).

**Table 2:** Univariate and multivariate analysis for cause-specific mortality in patients with type 2 diabetes mellitus

Outcomes	Quartiles of baPWV (cm/s)	Univariate		Multivariate	
		HR (95% CI)	*P* value	HR (95% CI)	P-value
All-cause mortality	Q1 (–1391)	Reference		Reference	
	Q2 (1392–1593)	1.09 (0.62–1.92)	0.760	0.89 (0.50–1.59)	0.703
	Q3 (1594–1829)	1.67 (1.00–2.80)	0.052	1.14 (0.66–1.96)	0.647
	Q4 (1830–)	5.39 (3.44–8.44)	< 0.001	2.55 (1.49–4.35)	0.001
Cardiovascular-mortality	Q1 (–1391)	Reference		Reference	
	Q2 (1392–1593)	4.52 (0.98–20.92)	0.054	3.60 (0.77–16.91)	0.104
	Q3 (1594–1829)	5.56 (1.23–25.08)	0.026	3.36 (0.72–15.73)	0.123
	Q4 (1830–)	14.89 (3.54–62.61)	< 0.001	5.57 (1.19–26.18)	0.030
Cancer-mortality	Q1 (–1391)	Reference		Reference	
	Q2 (1392–1593)	1.34 (0.47–3.86)	0.588	1.24 (0.43–3.62)	0.694
	Q3 (1594–1829)	2.02 (0.76–5.38)	0.160	1.80 (0.65–5.05)	0.261
	Q4 (1830–)	5.42 (2.25–13.02)	< 0.001	4.35 (1.57–12.03)	0.005
Other cause-mortality	Q1 (–1391)	Reference		Reference	
	Q2 (1392–1593)	0.54 (0.23–1.26)	0.154	0.41 (0.17–0.99)	0.046
	Q3 (1594–1829)	1.01 (0.49–2.07)	0.975	0.61 (0.28–1.30)	0.198
	Q4 (1830–)	4.12 (2.33–7.28)	< 0.001	1.59 (0.78–3.26)	0.207

Values are presented as odds ratio (95% confidence interval)

Multivariate model: after adjusting for age, sex, diabetes duration, BMI, HbA1c, SBP, GFR, smoking, and insulin

*baPWV* brachial-ankle pulse wave velocity, *HR* hazard ratio, *CI* confidence interval

**Fig. 2:**
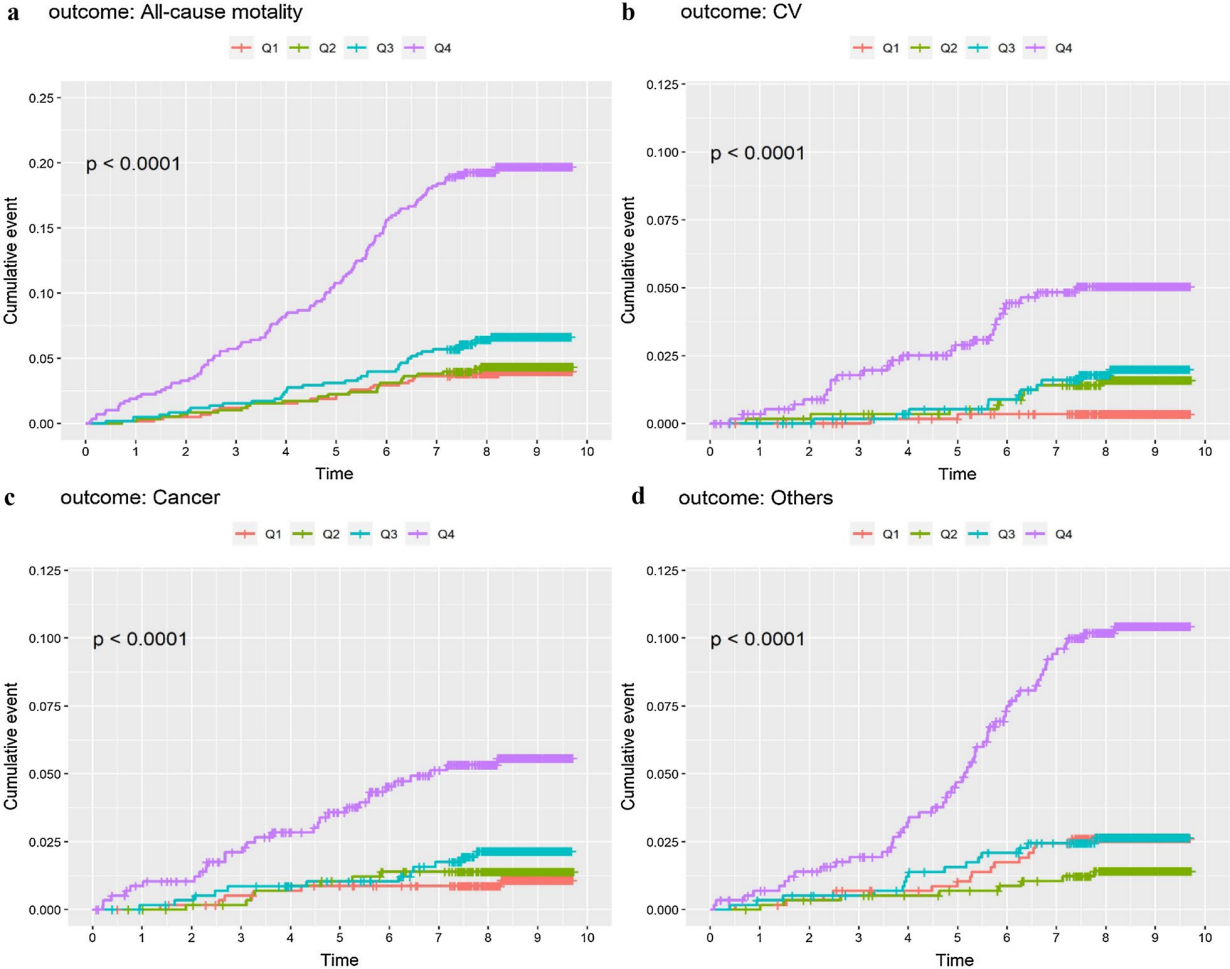
Cumulative incidence of all-cause and cause-specific mortality in patients with type 2 diabetes mellitus according to quartiles of baPWV (cm/s). **a** Cumulative incidence curve for all-cause mortality. **b** Cumulative incidence curve for cardiovascular-mortality. **c** Cumulative incidence curve for cancer-mortality. **d** Cumulative incidence curve for other cause-mortality. *baPWV* brachial-ankle pulse wave velocity, *CV* cardiovascular

Figure 2 presents significantly greater cumulative incidence of all-cause and cause-specific mortality for patients with type 2 diabetes. Across the quartile groups of baPWV, patients with the initial highest quartile of baPWV (Q4) had an increased risk for all-cause and cause-specific mortality (*P* < 0.001).

### Comparison of model performance with and without baPWV

We examined whether adding baPWV to the baseline model could improve the predictive power for the all-cause and cause-specific mortality. As shown in Table 3, the difference between the two models was not statistically significant for AUC for CV-mortality and other-cause mortality. However, adding baPWV to the baseline model containing confounding factors such as age, sex, diabetes duration, BMI, glycated hemoglobin, SBP, GFR, smoking, and insulin significantly improved NRI and IDI for all-cause mortality (NRI 0.487, 95% CI 0.347–0.627, *P* < 0.001; IDI 0.018, 95% CI 0.010–0.025, *P* < 0.001), CV-mortality (NRI 0.283, 95% CI 0.027–0.539, *P* = 0.030; IDI 0.004, 95% CI 0.001–0.007, *P *= 0.022), cancer-mortality (NRI 0.548, 95% CI 0.291–0.805, *P* < 0.001; IDI 0.013, 95% CI 0.002–0.023, *P* = 0.020), and other-cause mortality (NRI 0.510, 95% CI 0.329–0.691, *P* < 0.001; IDI 0.008, 95% CI 0.002–0.015, *P* = 0.009), respectively.

**Table 3:** Comparison of models with/without baPWV for the predication of mortality in type 2 diabetes mellitus

Model	All-cause mortality		Cardiovascular-mortality		Cancer-mortality		Other cause-mortality	
	95% CI	P-value	95% CI	P-value	95% CI	P-value	95% CI	P-value
Baseline								
AUC	0.770 (0.735–0.804)		0.788 (0.723–0.853)		0.785 (0.722–0.847)		0.749 (0.695–0.803)	
New								
AUC	0.788 (0.755–0.820)	0.008	0.799 (0.740–0.858)	0.377	0.807 (0.752–0.863)	0.043	0.782 (0.733–0.830)	0.011
NRI (categorical)	0.101 (0.055–0.147)	< 0.001	–0.076 (− 0.181–0.029)	0.155	–0.012 (− 0.106–0.082)	0.808	0.151 (0.042–0.261)	0.007
NRI (continuous)	0.487 (0.347–0.627)	< 0.001	0.283 (0.027–0.539)	0.030	0.548 (0.291–0.805)	<0.001	0.510 (0.329–0.691)	<0.001
IDI	0.018 (0.010–0.025)	< 0.001	0.004 (0.001–0.007)	0.022	0.013 (0.002–0.023)	0.020	0.008 (0.002–0.015)	0.009

Baseline model: age, sex, diabetes duration, BMI, HbA1c, SBP, smoking, and insulin. New model: baseline model + baPWV

*AUC* area under the receiver-operating characteristic curve, *baPWV* brachial-ankle pulse wave velocity, *CI* confidence interval, *NRI* net reclassification index, *IDI* integrated discrimination improvement

These finding suggested the additional prognostic value of baPWV for the prediction of mortality in type 2 diabetes mellitus.

## Discussion

The main finding in this long-term, prospective, multicenter cohort study of patients with type 2 diabetes was that a higher baPWV predicted an increased risk for all-cause and cause-specific mortality.

Concurring with previous reports, our results show that baPWV correlated with CV mortality in diabetes. A meta-analysis of longitudinal cohort studies reported a significant association between baPWV and CV mortality [22]. A few studies have reported that increased arterial stiffness, measured by baPWV or brachial pulse pressure, could predict mortality and CV events in subjects with diabetes [17, 23]. Several other prospective studies demonstrated that the optimal cutoff of baPWV for predicting CV mortality in a general population of elderly subjects was 1963 cm/s [24] and that for predicting a future CV event was 1800 cm/s [25]. However, both study follow-up durations were relatively short compared to our follow-up duration.

Yiming et al. demonstrated that the reference values of baPWV were significantly higher in subjects with diabetes than in normal subjects, after adjustment for age and mean blood pressure [26]. Zhang et al. reported a positive association between baPWV and the risk of new-onset diabetes in hypertensive patients [27]. It is possibly caused by advanced glycation end product formation with collagen cross-linking, oxidative stress, inflammatory process, and insulin resistance [28–31]. Multiple pharmacologic agents have been proposed to improve arterial stiffness. Empagliflozin induced improvement of arterial stiffness, endothelial dysfunction, and renal vascular stiffness by beneficial vascular effects via anti-inflammatory mechanisms [32–34]. Another anti-diabetic drug, an analog of human glucagon-like peptide 1, improves arterial stiffness by reducing oxidative stress [35]. In this study, baPWV values were higher in patients with type 2 diabetes than in the normal population of the same age categories (50 s and 60 s).

Regarding other causes of death, we observed a strong association between arterial stiffness and cancer prognosis in patients with type 2 diabetes. It is not yet clear whether the link between arterial stiffness and cancer prognosis is independent or dependent. Although we adjusted for clinical confounding factors, it is unclear whether there is a direct link between arterial stiffness and cancer because both share a number of factors related to common pathology. Recently, a retrospective observational study evaluated the role of arterial stiffness in the relationship between cancer and CVD [36]. They demonstrated that patients with malignancy had higher rates of adverse CV events and that stratifying by baPWV was valuable for adverse CV events in patients with malignancy. They further suggested that malignancy might contribute to the progression of arterial stiffness. Indeed, diabetes and cancer share many risk factors such as age, obesity, physical inactivity, and smoking [11], and diabetes is generally characterized by hyperglycemia and hyperinsulinemia, which may contribute to the proliferation of arterial smooth muscle and eventually lead to arterial stiffness [37]. Chronic hyperinsulinemia is also a significant factor explaining cancer initiation and progression in patients with diabetes, due to the tumorigenic effect of insulin [38]. Thus, hyperinsulinemia may be considered one of the factors that mediate between arterial stiffness and cancer outcomes in our study. Similarly, cancer-associated thrombosis could be another explanatory factor [39–42]. A previous study reports changes in the cause of death in Korean patients with type 2 diabetes over the periods 1990-1994 [43] and 2000–2004 [44]. In addition, even though the analysis has not been published, cancer became the most common cause of death, while CVD significantly decreased, in Korean patients with type 2 diabetes between 1990 and 2014 (1990–1994 CVD 37.6%, malignancy 4.7%; 2000–2004 CVD 30.6%, malignancy 21.9%; 2010–2014 CVD 11.6%, malignancy 38.5%) [45]. Thus, it is worthwhile discovering a means of predicting a cancer outcome, one of the main causes of death in diabetes patients.

Our study has some limitations. First, although a number of potential confounding factors, such as age, sex, diabetes duration, BMI, glycated hemoglobin, SBP, GFR, smoking, and insulin were controlled for in the multivariate regression analysis, other unrecognized confounding variables may exist. For example, we could not adjust for confounding lifestyle behaviors such as alcohol consumption, physical activity, and carbohydrate intake. In addition, we could not correct socio-economic status and educational level that are essential correction factors for other mortality analysis. Second, there was no non-diabetic control group. Third, two hospitals were excluded from the original eight hospitals due to the principal investigator’s relocation. It might add some bias to the analyses. Finally, PWV, which is a marker of arterial stiffness, was assessed only by baPWV. Although baPWV reflects arterial stiffness, carotid-femoral PWV is the gold standard for atherosclerosis measurement [14]. Despite these limitations, our study was conducted with a long-term, large-scale, multicenter prospective observational cohort, which represents significant strength. Furthermore, the observational period (i.e., a median follow-up period of 8.6 years) may have been sufficient for CVD and cancer outcomes to manifest.

## Conclusions

We found that a higher baPWV can predict all-cause and cause-specific mortality in subjects with type 2 diabetes. To our knowledge, our study is the first to use a long-term and large-scale prospective cohort to demonstrate the predictive role of baPWV for cancer prognosis independent of conventional risk factors in people with type 2 diabetes. The measurement of baPWV as a noninvasive tool for arterial stiffness is useful for assessing mortality risk assessment in patients with type 2 diabetes.

## Data Availability

The data of this study may be available on reasonable request to the corresponding author.

## Abbreviations

ABI: Ankle brachial index
baPWV: Brachial-ankle pulse wave velocity
CI: Confidence interval
CV: Cardiovascular
eGFR: Estimated glomerular filtration rate
HRs: Hazard ratios
hsCRP: High-sensitivity C-reactive protein
IDI: Integrated discrimination improvement
NRI: Net reclassification index
SD: Standard deviation
